# Electrophysiological and Molecular Features of Remdesivir-Induced Cardiac Toxicity in Male and Female Guinea Pigs

**DOI:** 10.3390/ijms27083685

**Published:** 2026-04-21

**Authors:** Chen Zhu, Kun Fu, Hu Wen, Guangqi Chen, Henggui Zhang

**Affiliations:** 1 Key Laboratory of Medical Electrophysiology, Ministry of Education and Medical Electrophysiological Key Laboratory of Sichuan Province, Collaborative Innovation Center for Prevention of Cardiovascular Diseases, Institute of Cardiovascular Research, Southwest Medical University, Luzhou 646099, China; 20234099120024@stu.swmu.edu.cn (C.Z.); 20230299120002@stu.swmu.edu.cn (K.F.); 20234099120033@stu.swmu.edu.cn (H.W.); 20244099120017@stu.swmu.edu.cn (G.C.); 2 Beijing Academy of Artificial Intelligence, Beijing 100084, China; 3 Biological Physics Group, Department of Physics and Astronomy, The University of Manchester, Manchester M13 9PL, UK

**Keywords:** cardiac toxicity, electrophysiology, mitochondrial dysfunction, remdesivir, sex difference

## Abstract

The global spread of COVID-19 led to the rapid authorization of remdesivir as the first antiviral therapy. However, accumulating clinical evidence has linked its use to cardiac adverse effects. Understanding the mechanisms underlying remdesivir-induced cardiotoxicity is critical for optimizing its clinical use and ensuring patient safety. This study investigates the electrophysiological and molecular features underlying remdesivir-induced cardiac toxicity in male and female guinea pigs, aiming to elucidate the sex-dependent differences in cardiac dysfunction and the role of mitochondria in mediating these effects. A cardiac injury model was established via intraperitoneal administration of remdesivir. In vivo telemetry and ex vivo electrocardiography were used for continuous monitoring of cardiac electrical activity, while optical mapping enabled the assessment of action potential parameters and conduction properties. The histopathological alterations and mitochondrial ultrastructure were examined by hematoxylin–eosin staining and transmission electron microscopy. ELISA and Western blot analyses were performed to explore the inflammatory signaling, apoptosis, and mitochondrial dynamics. Remdesivir induced distinct sex-specific patterns of cardiac toxicity. Compared with female guinea pigs, male guinea pigs had significantly more severe myocardial injury, which was characterized by extensive inflammatory cell infiltration, marked mitochondrial disruption, and a higher incidence of sustained ventricular tachyarrhythmia. Overall, remdesivir was associated with sex-dependent cardiac toxicity, accompanied by mitochondrial impairment and inflammatory activation. Male guinea pigs were more susceptible to electrophysiological instability and mitochondrial dysfunction. These findings highlight the importance of carefully evaluating remdesivir’s cardiac effects and support the need for individualized, sex-specific considerations in its clinical administration.

## 1. Introduction

The emergence of coronavirus disease 2019 (COVID-19) in late 2019 marked the beginning of an unprecedented global health crisis. Within months, the disease spread across continents, causing millions of infections and substantial mortality, overwhelming healthcare systems worldwide [1]. In response to this urgent threat, the United States Food and Drug Administration granted emergency use authorization for remdesivir, which is a broad-spectrum antiviral agent, in May 2020, making it the first clinical drug approved for COVID-19 treatment [2]. As a nucleoside analogue that inhibits viral RNA polymerase, remdesivir rapidly became a frontline therapy and was included in national and treatment guidelines [3]. Although remdesivir demonstrated efficacy in reducing viral replication and improving clinical outcomes, post-marketing and clinical observations have raised concerns on its cardiac safety [4]. Accumulating reports have indicated associations between its use and adverse cardiac events, such as sinus bradycardia, QT prolongation, cardiac arrest, and asystole [5]. Despite its continued clinical use, mechanistic studies on remdesivir-induced cardiotoxicity remain limited, highlighting the need for deeper investigation into its cardiotoxic impacts.

Epidemiological investigations during the COVID-19 pandemic revealed sex-specific differences in both disease susceptibility and clinical outcomes. Male patients consistently exhibited greater disease severity [6], higher rates of intensive care unit admission [7], and increased mortality [8,9], when compared with female patients. These disparities appear to have resulted from the interplay of multiple factors. Physiologically, males express higher levels of angiotensin-converting enzyme 2, which is the primary receptor that mediates SARS-CoV-2 entry into host cells [10,11]. Immunologically, males are more susceptible to hyperinflammatory responses, including cytokine storm syndromes, which contribute to more severe clinical progression [12]. Furthermore, behavioral and lifestyle factors, such as higher rates of smoking and alcohol consumption, may further exacerbate disease severity in males [13]. Although both age and sex independently influence COVID-19 outcomes, present evidence suggests no significant interactions between these. Such disparities highlight the need for effective therapies, and remdesivir provides a promising way to inhibit viral replication and improve clinical outcomes.

Clinical and immunological analyses have further illustrated the biological basis of these sex-related disparities. Male patients generally exhibit higher susceptibility, poorer prognosis, weaker immune responses, and a greater burden of comorbidities [14,15]. In contrast, female patients typically exhibit stronger innate and adaptive immunity, which are often associated with more favorable clinical outcomes [16]. However, this immunological advantage can lead to excessive inflammation and tissue injury in some cases [17]. These differences are partially modulated by sex hormones [18,19] and genetic factors [20]. Consequently, despite comparable exposure risks, the overall clinical trajectory of COVID-19 substantially differs between sexes. Recognizing this, the World Health Organization urged (early in the pandemic) the systematic collection and reporting of sex-disaggregated data on infection rates, hospitalization, and mortality [21]. Such data are essential for elucidating the biological determinants of viral infection and providing a scientific basis for developing tailored therapeutic interventions. Investigating how remdesivir affects different patient groups is key to optimizing treatment strategies and improving outcomes for both sexes.

Given these clinical and epidemiological insights, it is plausible that sex also influences pharmacological responses, including the cardiac safety profile of remdesivir. Although remdesivir has been widely used in the treatment of COVID-19, emerging clinical observations suggest variability in cardiac adverse events among patients. In addition, sex-specific differences in its potential cardiotoxic effects, such as QT prolongation and sinus bradycardia, remain poorly understood. Therefore, the present study aimed to investigate the electrophysiological and molecular features underlying remdesivir-induced cardiac toxicity in male and female guinea pig models.

## 2. Results

### 2.1. Remdesivir-Induced Behavior Changes Differed Between Sexes

A total of 32 guinea pigs (16 male and 16 female guinea pigs) were randomly assigned to the control (saline) and model (remdesivir-treated) groups (*n* = 8 per sex per group). Both groups received intraperitoneal injections for five consecutive days, followed by a 3-day observation period. During treatment, remdesivir-treated animals exhibited reduced food intake, decreased locomotor activity, and diminished responsiveness, when compared with controls. One male guinea pig in the model group died on the fifth day of treatment, indicating male-specific intolerance, while no mortality occurred in female guinea pigs. After discontinuation of treatment, all surviving animals in both sexes showed full recovery of behavior and appetite within three days, accompanied by normal activity and body weight gain. No further deaths occurred during the post-treatment observation period.

### 2.2. Males Exhibited More Severe Inflammatory Injury in the Heart After Remdesivir Exposure

**Figure 1:**
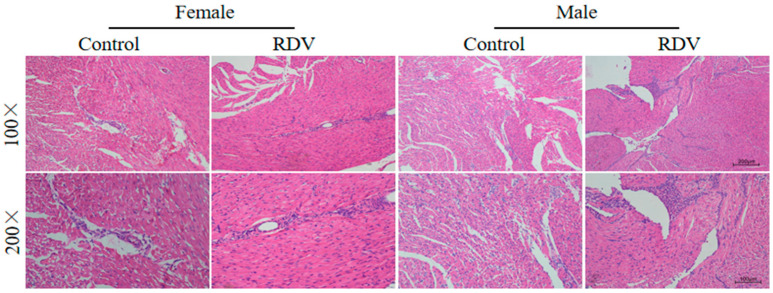
Histopathological changes in atrial tissues following remdesivir exposure. Representative H&E staining images of atrial tissues in male and female guinea pigs at 100× and 200× magnifications.

The gross examination revealed no apparent morphological differences in heart size, chamber dimensions, or epicardial surface appearance among the groups. However, the H&E staining revealed clear sex-dependent differences in inflammatory and structural injury patterns (Figure 1). In female guinea pigs, remdesivir administration induced only mild, focal inflammatory infiltration that localized mainly around the perivascular regions, with preserved overall myocardial fiber alignment and integrity. No evident necrosis or deformation of cardiomyocytes was observed. In contrast, male guinea pigs exhibited diffuse myocarditis characterized by extensive neutrophil-dominated inflammatory infiltration (accounting for approximately 70–80% of infiltrating cells) within both the myocardial interstitium and subendocardial regions. Marked cytoplasmic swelling, enhanced eosinophilia, loss of cross-striations, and focal myofibrillar condensation were also observed. These findings indicate more severe myocardial inflammation and necrosis occurred in male guinea pigs, when compared with female guinea pigs.

### 2.3. Mitochondrial Damage Is Prominent in Male Cardiomyocytes

**Figure 2:**
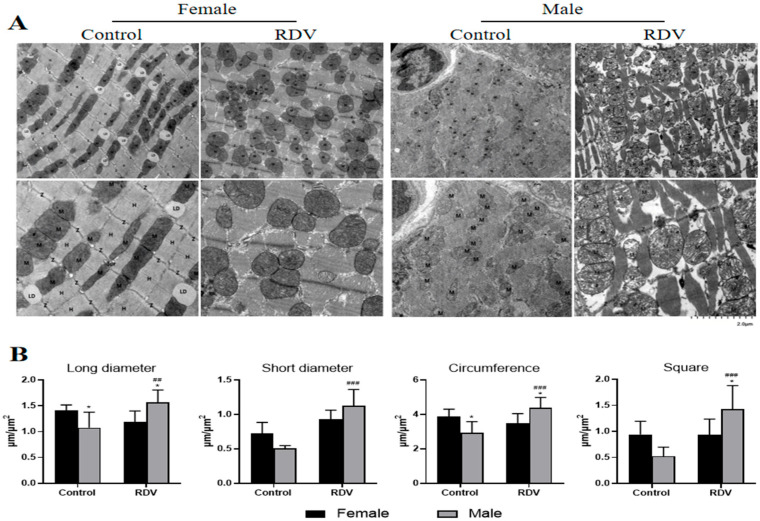
Ultrastructural alterations of cardiomyocyte mitochondria observed by TEM. (**A**) The TEM images illustrate the mitochondrial morphology in atrial cardiomyocytes. Bar = 2 μm. (**B**) The long diameter, short diameter, circumference, and square of each group were quantified. * *p* < 0.05 vs. female guinea pigs; ## *p* < 0.01, ### *p* < 0.001 vs. controls in the same sex groups.

The TEM results revealed distinct ultrastructural differences between sexes following remdesivir exposure (Figure 2). In control animals, myocardial cells appeared structurally intact with well-aligned myofibrils and normal mitochondrial morphology. In male guinea pigs of the model group, cardiomyocytes presented with moderate swelling, disorganized myofibrillar arrangement, frequent rupture and dissolution of myofibril bundles, and indistinct A- and H-bands. Furthermore, the mitochondria were moderately swollen and irregularly distributed, with disrupted cristae and localized matrix rarefaction. Moreover, occasional myelin-like structures were observed, while autophagic vesicles were absent. In contrast, female model animals maintained a relatively intact cellular and mitochondrial morphology. Myofibrils remained well-organized with clear Z-lines and H-bands, and the mitochondria presented with uniform matrices with largely preserved cristae. Furthermore, mild sarcoplasmic reticulum dilation and an increased number of lipid droplets were observed, suggesting compensatory metabolic adaptation, rather than overt structural damage.

### 2.4. Remdesivir Triggers Stronger Inflammatory Responses and Energy Impairment in Males

**Figure 3:**
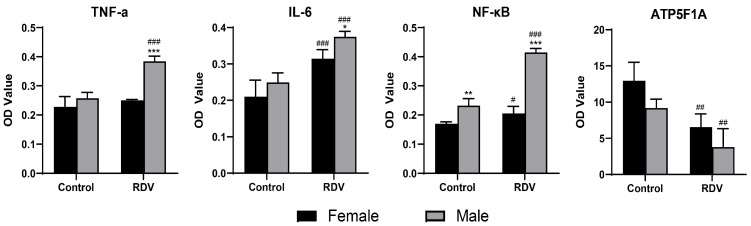
Sex-dependent differences in inflammatory cytokines and ATP5F1A levels. The bar graphs show the concentrations of TNF-α, IL-6 and NF-κB, and ATP5F1A levels in atrial tissues, as detected by ELISA. * *p* < 0.05, ** *p* < 0.01, *** *p* < 0.001 vs. females; # *p* < 0.05, ## *p* < 0.01, ### *p* < 0.001 vs. controls in the same sex groups (*n* = 6 per group).

**Figure 4:**
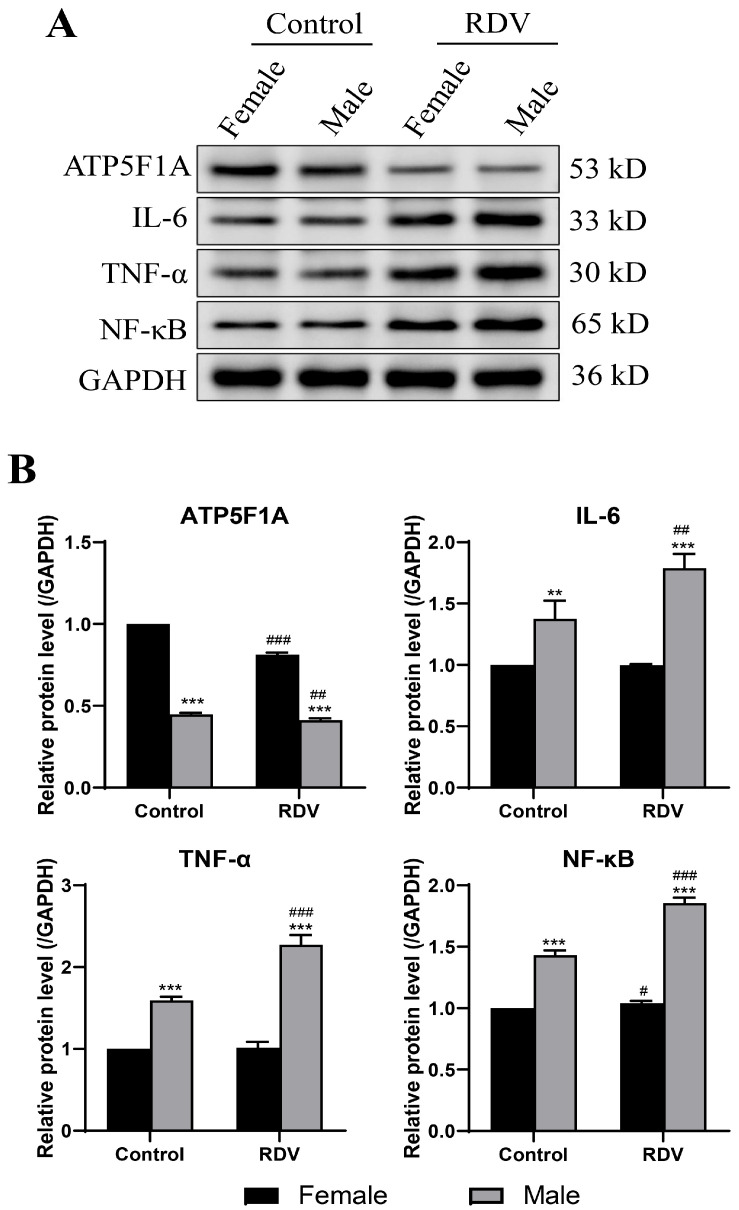
Western blot analysis of inflammatory protein expression in atrial tissues. (**A**) Representative Western blots and quantitative analysis of IL-6, TNF-α, NF-κB and ATP5F1A expression in control, and remdesivir-treated male or female hearts. (**B**) The protein levels were normalized to GAPDH. ** *p* < 0.01, *** *p* < 0.001 vs. females; # *p* < 0.05, ## *p* < 0.01, ### *p* < 0.001 vs. controls in the same sex groups.

The ELISA analyses (Figure 3) revealed that the expression levels of proinflammatory cytokines IL-6, TNF-α and NF-κB were significantly elevated in remdesivir-treated animals, when compared with controls. The sex-based comparison further revealed that male guinea pigs exhibited markedly higher cytokine levels, when compared to female guinea pigs. The quantification of ATP5F1A exhibited a pronounced decline in both sexes, with males having a greater reduction (−45%), when compared with females (−30%), indicating the more severe impairment of cardiac energy metabolism in males. The Western blot analysis results (Figure 4) further confirmed the activation of inflammatory signaling. The protein levels of IL-6, TNF-α and NF-κB were 2–3 times higher in the model group, when compared with controls, and these were approximately 1.8 times higher in male guinea pigs, when compared with female guinea pigs. Collectively, these molecular findings demonstrate that remdesivir induces stronger inflammatory activation and mitochondrial dysfunction in a sex-dependent manner, with males being more susceptible to cardiac injury.

### 2.5. Sex Differences in In Vivo Electrophysiological Responses to Remdesivir

**Figure 5:**
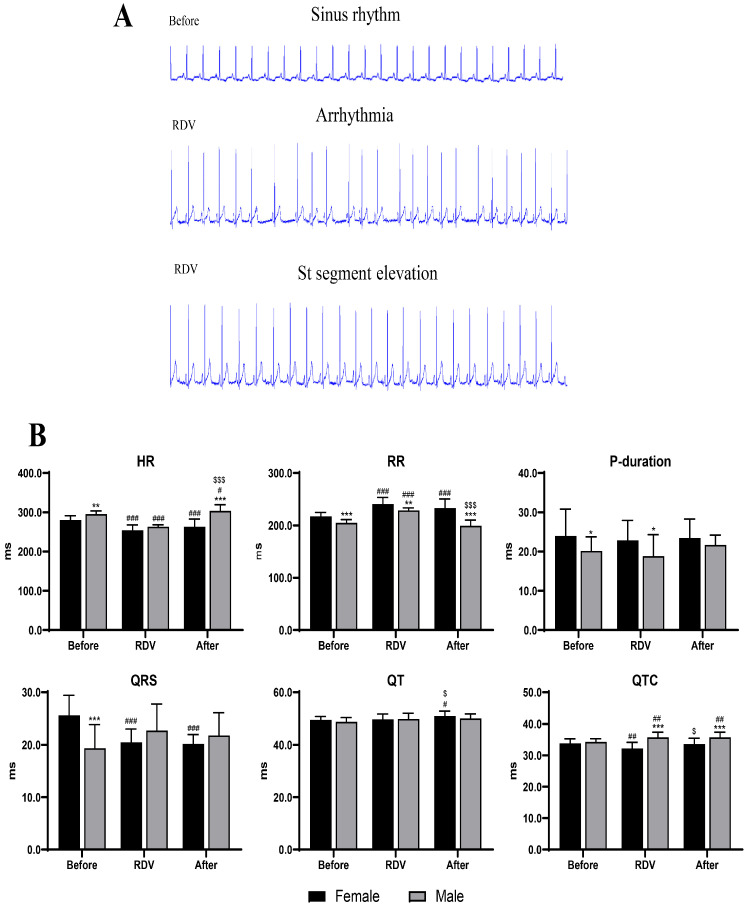
The in vivo ECG shows changes in HR during remdesivir treatment. (**A**) Representative ECG waveforms showing the sinus rhythm, arrhythmic episodes, and ST-segment elevation in male and female guinea pigs. (**B**) Quantitative analyses of HR, RR interval, P-wave duration, QRS duration, QT interval, and QTc are shown. The QT interval was corrected for HR using Bazett’s formula (QTc = QT/√RR).* *p* < 0.05, ** *p* < 0.01, *** *p* < 0.001 vs. female guinea pigs; # *p* < 0.05, ## *p* < 0.01, ### *p* < 0.001 vs. before in the same sex groups; $ *p* < 0.05, $$$ *p* < 0.001 vs. remdesivir in the same sex groups.

The continuous ECG monitoring revealed significant alterations in cardiac rhythm during and after remdesivir treatment (Figure 5). Both sexes had prolonged QT intervals and RR intervals, accompanied by a significant reduction in heart rate (HR), when compared with baseline. Notably, the magnitude of QT prolongation was approximately 25% greater in male guinea pigs, and the RR intervals increased substantially more. The recovery of cardiac rhythm was slower in male guinea pigs, with residual QT prolongation and bradycardia persisting on day three of the post-treatment period. These results indicate that males experience greater electrophysiological instability and delayed recovery following remdesivir exposure.

**Figure 6:**
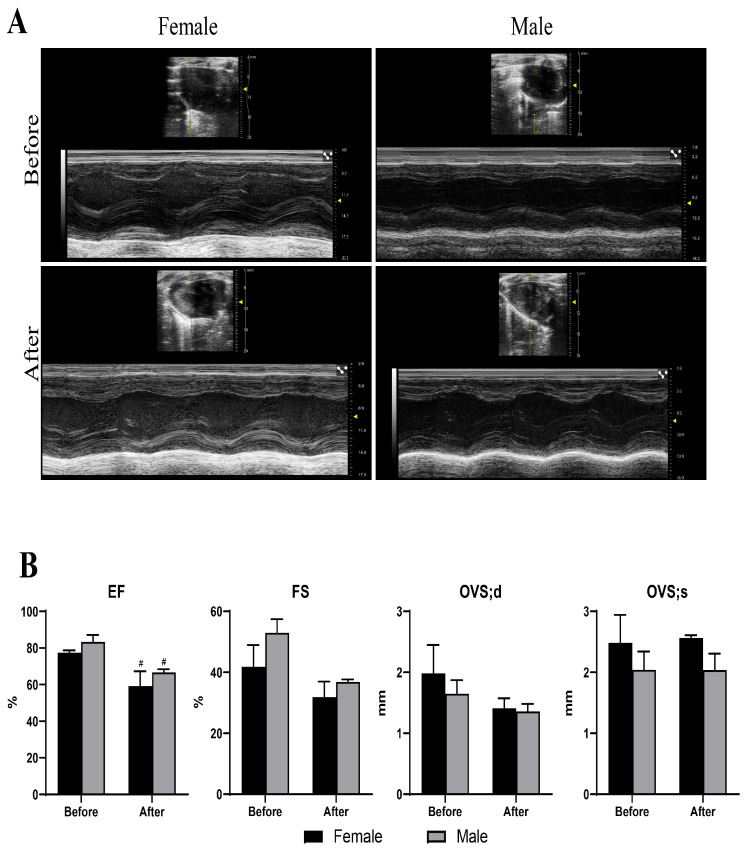
Echocardiographic assessment of cardiac function. (**A**) Representative echocardiographic images. The yellow line is the M-mode measurement line, and the arrow indicates the midpoint of the measurement range. (**B**) Quantitative analysis of EF, FS, IVS;d and IVS;s. # *p* < 0.05 vs. controls in the same sex groups.

The echocardiographic analysis results (Figure 6) further revealed a significant reduction in EF following remdesivir administration, while the fractional shortening and interventricular septal thickness during diastole and systole exhibited no significant changes. The decrease in EF indicates impaired cardiac contractile function, suggesting that the remdesivir treatment might have induced mild structural or functional myocardial injury.

### 2.6. Ex Vivo Findings Confirm the Male-Predominant Electrical Instability

**Figure 7:**
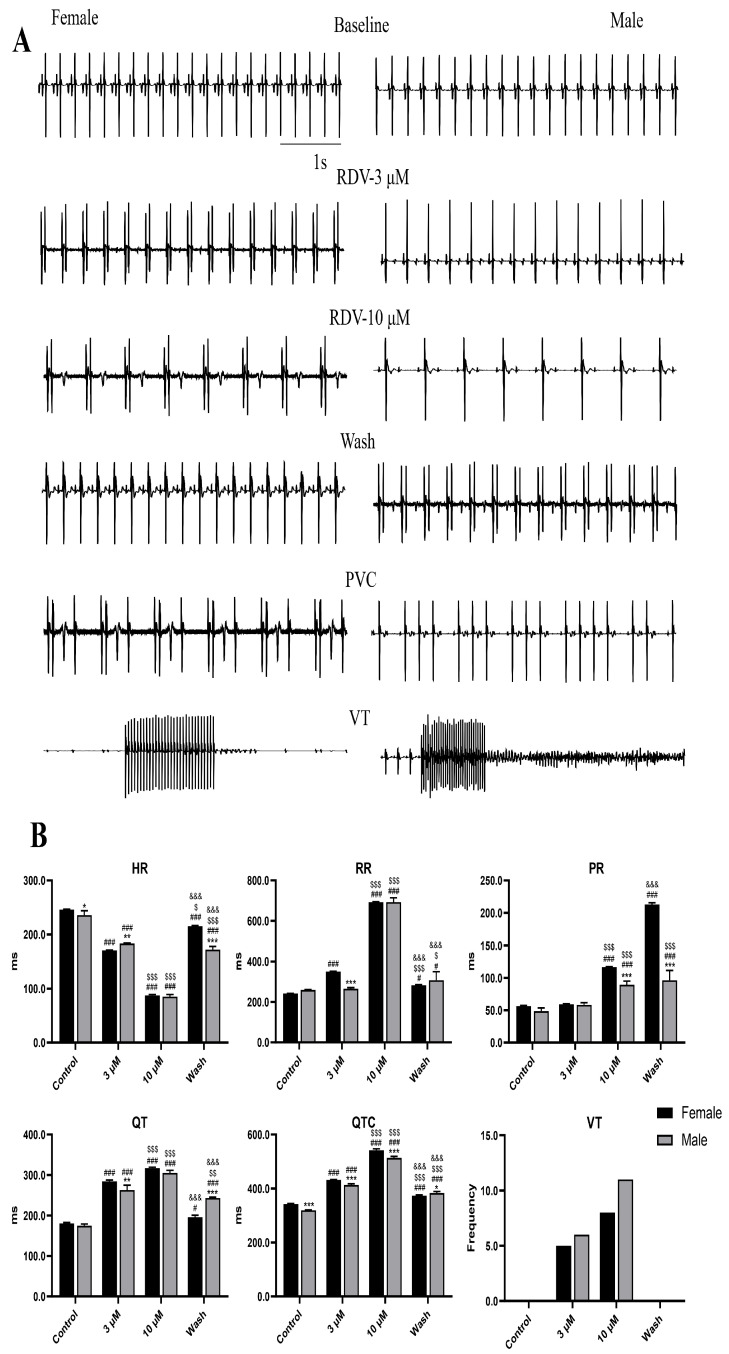
Ex vivo ECG of Langendorff-perfused hearts demonstrating sex-dependent arrhythmic responses. (**A**) Representative ECG traces and (**B**) quantitative data for heart rate HR, RR, PR, QT, QTc, and VT durations across four experimental groups: control, 3 µM remdesivir, 10 µM remdesivir, and washout. The QT interval was corrected for HR using Bazett’s formula (QTc = QT/√RR). * *p* < 0.05, ** *p* < 0.01, *** *p* < 0.001 vs. female guinea pigs; # *p* < 0.05, ### *p* < 0.001 vs. controls in the same sex groups; $ *p* < 0.05, $$ *p* < 0.01, $$$ *p* < 0.001 vs. 3 µM remdesivir in the same sex groups; &&& *p* < 0.001 vs. 10 µM remdesivir in the same sex groups.

The ex vivo Langendorff-perfused heart recordings (Figure 7) revealed dose-dependent electrophysiological changes induced by remdesivir, which were consistent with the in vivo findings. For the RR intervals, no significant difference was observed between the control and 3 μM remdesivir groups in male guinea pigs. For the PR intervals, both male and female hearts had no significant differences between the control and 3 μM remdesivir groups. In contrast, HR, QT, and QTc exhibited significant, concentration-dependent changes across groups. The sex-specific comparisons further revealed that males exhibited more pronounced and dynamic electrophysiological changes in response to remdesivir exposure and washout. These differences were particularly evident during drug challenge and the recovery phase, indicating altered electrical adaptation and delayed normalization in males compared with females. Collectively, these results indicate the dose- and sex-dependent effect of remdesivir on cardiac conduction and rhythm, with males exhibiting greater electrophysiological vulnerability.

### 2.7. Sex- and Dose-Dependent Effects of Remdesivir on Cardiac Action Potentials

**Figure 8:**
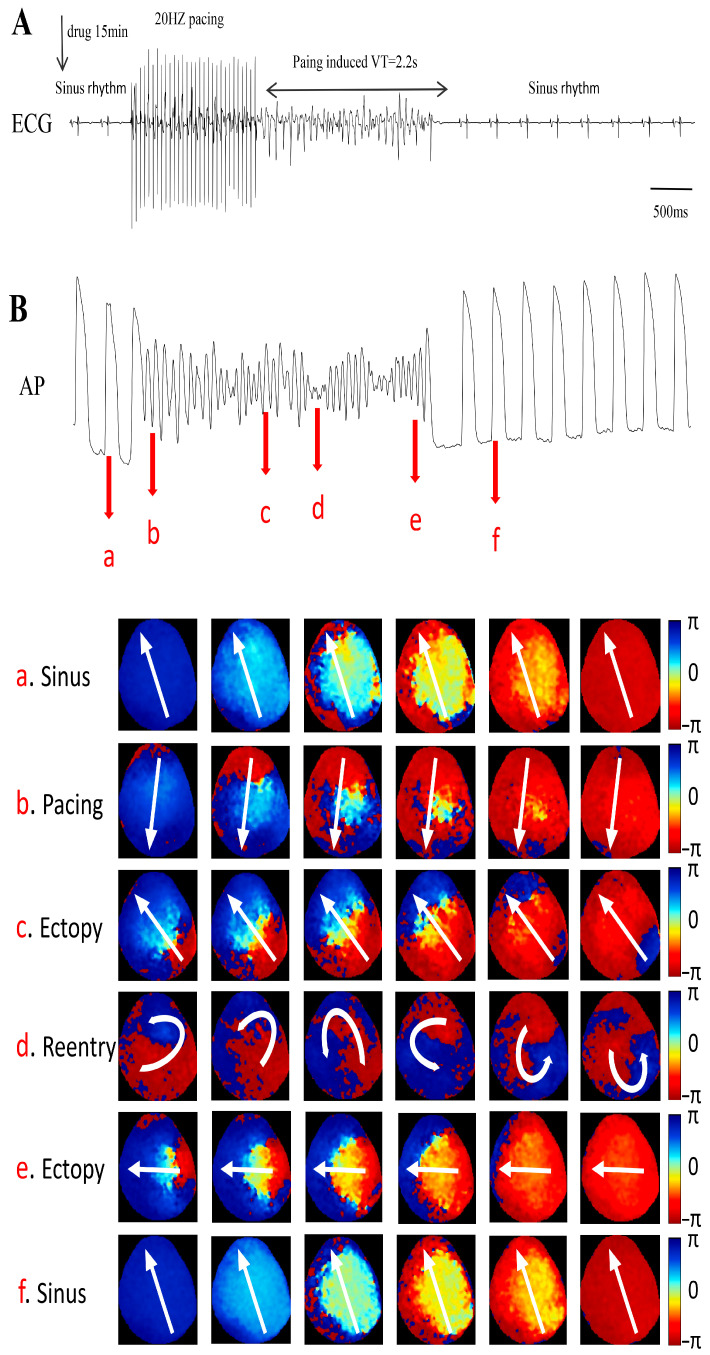
Optical mapping analysis of remdesivir-induced ventricular tachyarrhythmias. (**A**,**B**) ECG, AP map and phase map of conduction. The white arrow indicates the direction of conduction. ectopy and Reentry during the occurrence of the arrhythmia in both male and female guinea pigs.

The optical mapping experiments provided a detailed visualization of remdesivir-induced electrophysiological changes in both the whole-heart and atrial preparations (Figure 8). At 3 μM of remdesivir, male guinea pigs developed significant ventricular tachyarrhythmias, while female guinea pigs maintained a stable rhythm. For the action potential duration (APD50), neither male nor female guinea pigs presented with a significant difference between the 3 μM remdesivir and washout groups, while all other group comparisons exhibited significant changes. For APD80, female guinea pigs had no significant differences between the 10 μM remdesivir and washout groups, while male guinea pigs again had no differences between the 3 μM remdesivir and washout groups. All remaining comparisons were significantly different. At 10 μM of remdesivir, male guinea pigs exhibited a marked decrease in HR and failed to return to baseline rhythm during the recovery phase, while female guinea pigs presented with partially restored normal electrical activity.

**Figure 9:**
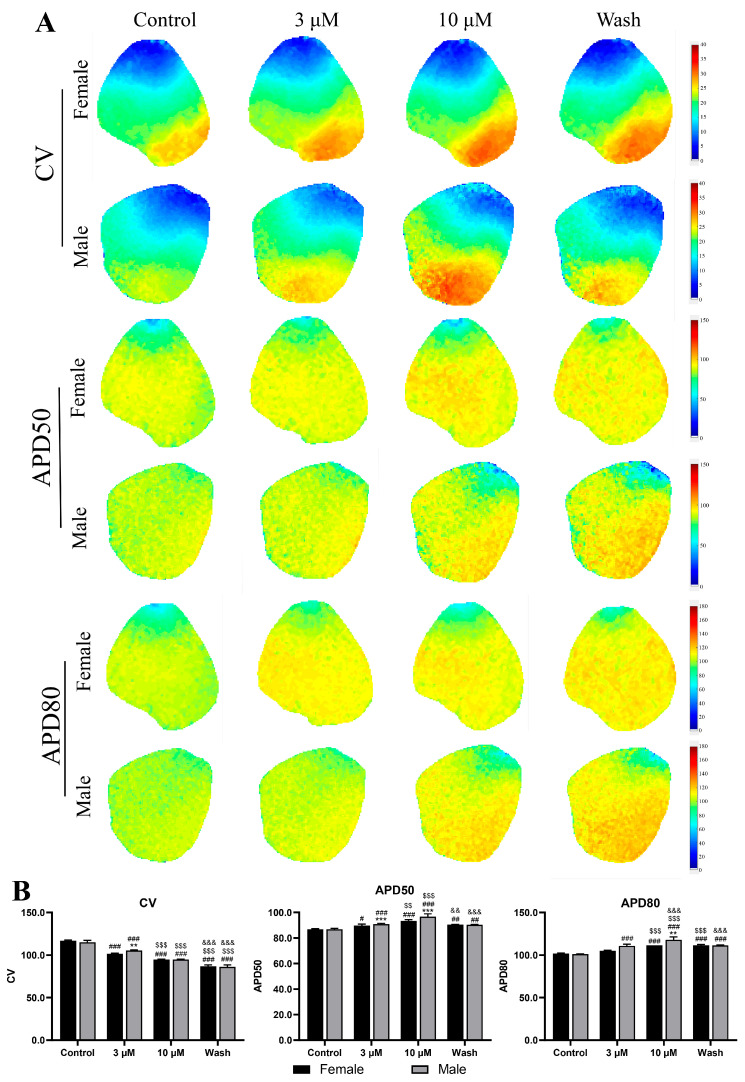
Comparison of atrial electrophysiological parameters between sexes by optical mapping. (**A**) The optical maps depict the CV and action potential duration at 50% and 80% repolarization (APD_50_ and APD_80_). (**B**) The quantitative analysis of each parameter. ** *p* < 0.01, *** *p* < 0.001 vs. female guinea pigs; # *p* < 0.05, ## *p* < 0.01, ### *p* < 0.001 vs. controls in the same sex groups; $$ *p* < 0.01, $$$ *p* < 0.001 vs. 3 µM remdesivir in the same sex groups; && *p* < 0.01, &&& *p* < 0.001 vs. 10 µM remdesivir in the same sex groups.

The analysis of action potential parameters (Figure 9) revealed prolonged activation time and increased action potential duration at 50% (APD50) and 80% repolarization (APD80) in male guinea pigs, when compared with female guinea pigs. Specifically, the atrial optical mapping revealed that for male guinea pigs, activation time increased by 21.3 ± 3.2% (vs. 12.5 ± 2.1% in female guinea pigs), APD50 increased by 18.7 ± 2.8% (vs. 9.3 ± 1.9% in female guinea pigs), and APD80 increased by 23.5 ± 3.1% (vs. 14.2 ± 2.3% in female guinea pigs). These data collectively demonstrate that remdesivir exerts dose-dependent and sex-dependent effects on cardiac electrical activity, with males showing greater prolongation of repolarization and reduced electrophysiological resilience.

### 2.8. Calcium Imaging in Atrial Tissues Following Remdesivir Exposure

**Figure 10:**
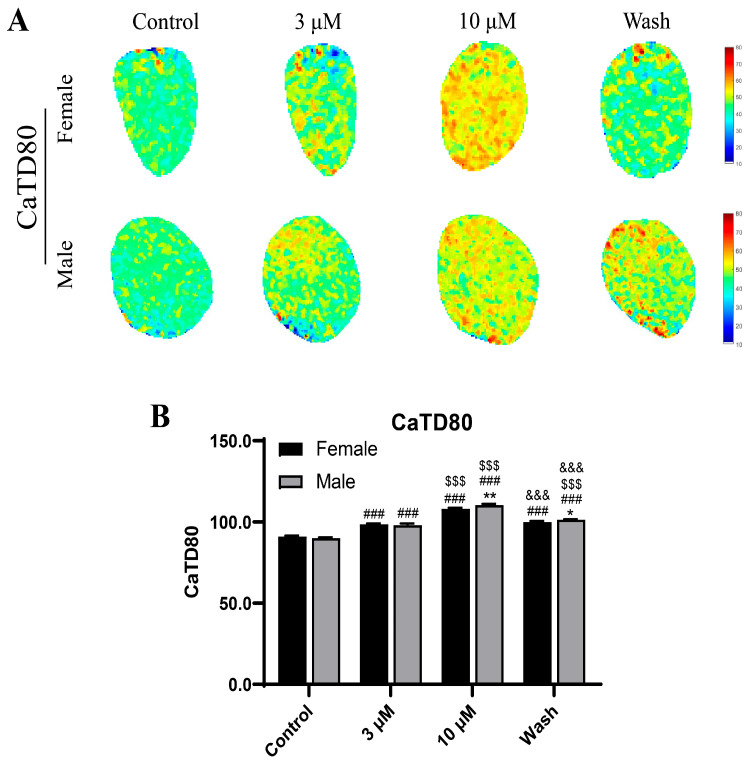
Calcium imaging and analysis in atrial tissues. (**A**) Representative pseudocolor calcium transient maps illustrating alterations in Ca^2+^ handling at increasing concentrations of remdesivir (0, 3 and 10 μM) and after washout. (**B**) The quantitative analysis of CaTD80 under the same conditions.* *p* < 0.05, ** *p* < 0.01 vs. female guinea pigs; ### *p* < 0.001 vs. controls in the same sex groups; $$$ *p* < 0.001 vs. 3 µM of remdesivir in the same sex groups; &&& *p* < 0.001 vs. 10 µM of remdesivir in the same sex groups (*n* = 6 per group).

The calcium imaging (Figure 10) revealed concentration-dependent alterations in Ca^2+^ handling following remdesivir exposure. At 3 μM of remdesivir, male guinea pigs had a modest prolongation of calcium transient duration, while female guinea pigs maintained relatively stable Ca^2+^ dynamics. At 10 μM of remdesivir, both sexes presented with pronounced slowing of calcium reuptake, but the effect was significantly greater in male guinea pigs, which is consistent with the prolonged electrical repolarization. The quantitative analysis of CaTD80, which is defined as the duration for the calcium transient to decay to 80% of its peak amplitude, confirmed these observations. In female guinea pigs, no significant difference was detected between the 3 μM remdesivir and washout groups, while all other comparisons had significant differences. These findings indicate that remdesivir disrupts calcium homeostasis and prolongs repolarization in a dose- and sex-dependent manner, with males exhibiting greater susceptibility to excitation–contraction coupling dysfunction.

## 3. Discussion

**Figure A1:**
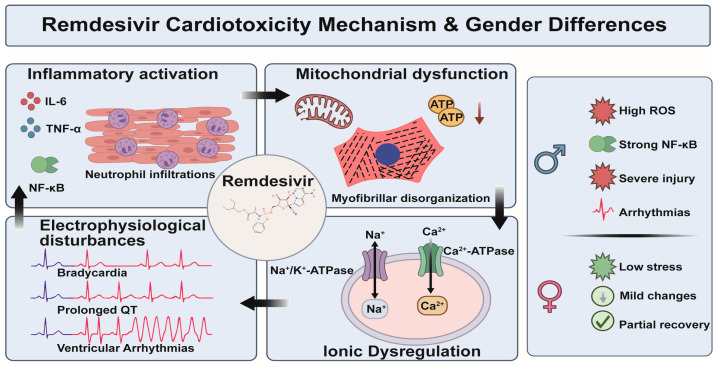
Schematic of sex-dependent remdesivir cardiotoxicity driven by inflammation, mitochondrial dysfunction, ionic imbalance, and electrophysiological instability.

In the present study, remdesivir administration was associated with pronounced, sex-dependent cardiac toxicity in guinea pigs. Male animals presented with more severe myocardial injury, which was characterized by diffuse myocarditis, extensive neutrophil infiltration, and focal myocyte necrosis. These structural changes were accompanied by mitochondrial dysfunction, including swelling, cristae disruption, and myofibrillar disorganization, which collectively contributed to impaired cardiac energy metabolism. The electrophysiological assessments revealed marked bradycardia, prolonged QT and RR intervals, and sustained ventricular arrhythmias in male guinea pigs, indicating heightened vulnerability. In contrast, female guinea pigs had milder cardiac effects with a largely preserved myocardial and mitochondrial structure, and only transient electrophysiological disturbances. Overall, these results highlight the robust sex-specific disparity in remdesivir-induced cardiotoxicity, suggesting that male guinea pigs are more susceptible to both structural and functional cardiac perturbations (Figure A1).

Guinea pigs were selected as the experimental model for cardiac electrophysiology due to its close similarity to human cardiac electrical properties. First, guinea pig ventricular myocytes exhibit a pronounced phase 2 plateau in its action potentials, which closely resembles the human ventricular action potential, unlike rats or mice, which lack a distinct plateau phase [22]. Second, the ventricular repolarization in guinea pigs primarily depends on the rapid delayed rectifier potassium current (IKr) and slow delayed rectifier potassium current, which is a mechanism highly comparable to humans. In contrast, murine ventricular repolarization relies mainly on transient outward potassium current, resulting in electrophysiological differences from humans [22,23]. Therefore, guinea pigs provide a more appropriate model, when compared to other rodents, for studying human-relevant drug-induced cardiotoxicity, especially for compounds that affect repolarization.

The concentrations of remdesivir used in the ex vivo experiments (3 μM and 10 μM) were selected based on both the clinical pharmacokinetic data and prior experimental evidence. A pharmacokinetic study revealed that after intravenous administration of the standard therapeutic dose of remdesivir in COVID-19 patients, the peak plasma concentration of the parent compound ranged from approximately 2.7 to 7.3 μM [24]. In animal models, comparable dosing regimens can produce plasma concentrations in the range of approximately 0.5–4.0 μM [25]. Therefore, the concentrations used in the present study (3 μM and 10 μM) cover and slightly exceed the clinically observed pharmacologically active range. Furthermore, this concentration range was validated in the previous study [26] conducted by the investigators, where this reliably induced electrophysiological cardiotoxicity phenotypes consistent with clinical observations, including sinus bradycardia and QT interval prolongation. The use of these experimentally established concentrations enabled the investigation of the underlying electrophysiological and molecular mechanisms of remdesivir-induced cardiac toxicity under pharmacologically relevant conditions [27].

Mitochondria are central regulators of cardiac bioenergetics, maintaining the ATP production required for contractile activity, ion homeostasis, and electrophysiological stability [28]. Impaired mitochondrial function has been implicated in a variety of drug-induced cardiotoxicities, including those caused by antivirals (e.g., zidovudine) and chemotherapeutics (e.g., doxorubicin) [29]. In the present study, the remdesivir treatment significantly reduced the atrial ATP5F1A levels, indicating compromised energy metabolism. The energy deficit likely impaired the ATP-dependent ion pumps, such as Na^+^/K^+^-ATPase, disrupting the ionic gradients across the sarcolemma [30]. This resulting imbalance may have contributed to the mitochondrial matrix swelling, cytoplasmic edema, and further deterioration of cardiomyocyte integrity [31]. The TEM results revealed morphological alterations that are consistent with mitochondrial dysfunction, including cristae disorganization and myelin-like structures, supporting the central role for mitochondrial compromise in mediating remdesivir-induced cardiac injury.

The disruption of mitochondrial dynamics, particularly the balance between fusion and fission, is a key mechanism underlying drug-induced cardiac dysfunction [32]. Excessive fission or abnormal fusion fragments mitochondria, impairs energy distribution, and facilitates reactive oxygen species (ROS) accumulation, which further damages cellular components (e.g., proteins, lipids, and nucleic acids) [33]. In remdesivir-treated male guinea pigs, the mitochondrial swelling, disorganized myofibrils, and reduced ATP5F1A levels were consistent with disturbed mitochondrial dynamics. These alterations likely contributed to the observed electrophysiological abnormalities by compromising ion homeostasis and reducing cardiac excitability. Notably, these findings are in line with previous reports that revealed that antiviral agents can induce mitochondrial dysfunction, prolong action potentials, and increase susceptibility to arrhythmogenesis [34,35].

The mechanisms underlying remdesivir-induced QT interval prolongation remains undefined. In a guinea pig model, Pilote et al. reported that remdesivir induced bradycardia and mild QTc prolongation. However, they did not observe the direct blockade of the human ether-a-go-go-related gene (hERG) channel, suggesting that the QT-prolonging effect might involve indirect mechanisms. In contrast, the previous work conducted by the investigators using patch-clamp electrophysiology revealed that remdesivir significantly inhibited the hyperpolarization-activated cyclic nucleotide-gated channel 4 (HCN4)-mediated If current in sinoatrial node cells (>40% inhibition), and the hERG-mediated IKr current, with inhibition rates of 32.7% and 34.9% at 3 μM and 10 μM, respectively [26]. These inhibitory effects may account for the sinoatrial node dysfunction and QT prolongation. In the present study, male guinea pigs exhibited approximately 25% more pronounced QT prolongation, when compared to female guinea pigs, which was accompanied by more severe mitochondrial dysfunction (ATP5F1A reduction of 45% vs. 30% in females) and stronger inflammatory activation, including significantly elevated IL-6, TNF-α, and NF-κB levels. The investigators speculate that mitochondrial ATP depletion may impair the activity of ATP-dependent ion transporters, such as Na^+^/K^+^-ATPase and Ca^2+^-ATPase, thereby exacerbating the repolarization delay associated with IKr inhibition. In addition, inflammatory signaling may further suppress potassium channel function, and contribute to electrical instability [36]. The protective effects of estrogen on hERG and HCN4 channel function may partly explain the milder phenotype observed in female animals. Taken together, these findings suggest that the remdesivir-induced QT prolongation likely resulted from the combined effects of the direct ion-channel inhibition and sex-specific mitochondrial–inflammatory cascades.

Clinical data from COVID-19 have revealed that male patients experience greater disease severity and mortality [37], and are frequently accompanied by higher incidences of myocarditis, arrhythmias, and elevated cardiac injury biomarkers, when compared with female patients [38]. In the present study, male guinea pigs presented with more severe electrophysiological disturbances in both the in vivo and ex vivo models, which were characterized by bradycardia, prolonged QT and RR intervals, and sustained ventricular arrhythmias. In contrast, female guinea pigs presented with milder and transient electrical alterations, with better preserved mitochondrial integrity. These sex differences may reflect the multiple biological factors that modulate cardiac resilience [39]. Males express higher levels of angiotensin-converting enzyme 2, which is the primary receptor that mediates the SARS-CoV-2 entry into host cells, and may increase susceptibility to viral- or drug-induced cardiac stress [40]. In addition, males are more prone to hyperinflammatory responses, including elevated cytokine production, potentially exacerbating the oxidative stress and mitochondrial dysfunction [41]. Hormonal and genetic factors further contribute to cardiac resilience. For instance, estrogen exerts multifaceted cardioprotective effects by enhancing mitochondrial biogenesis, stabilizing mitochondrial membrane potential, and attenuating oxidative stress and inflammation [18,28,42]. Conversely, testosterone can promote mitochondrial ROS generation and amplify apoptotic signaling, potentially increasing cardiac vulnerability in males under pharmacological or pathological stress [20,43]. Furthermore, the presence of two X chromosomes in females provide a genetic advantage through the expression of immune- and metabolism-related genes, such as *TLR7* and *FOXP3*, which help maintain mitochondrial and inflammatory homeostasis [20]. Together, these mechanisms may explain the male-specific susceptibility to remdesivir-induced cardiac injury observed in the present study. The present findings align with the clinical observations in COVID-19 patients, wherein males are more prone to cardiac stress and adverse outcomes [44], supporting that sex-dependent biological mechanisms influence both viral pathophysiology and drug-induced cardiotoxicity.

The molecular, structural, and electrophysiological analyses further indicated that inflammatory activation plays a central role in remdesivir-induced cardiac injury. In male guinea pigs, the elevated expression of IL-6, TNF-α, and NF-κB was accompanied by diffuse inflammatory infiltration, mitochondrial swelling, and myofibrillar disorganization. These findings suggest that inflammation-induced oxidative and metabolic stress exacerbate mitochondrial injury and disrupt excitation–contraction coupling [45,46]. In addition, the present calcium imaging experiment revealed that remdesivir perturbs intracellular calcium handling, and delays repolarization in a dose- and sex-dependent manner, with male guinea pigs showing greater vulnerability to excitation–contraction coupling dysfunction. These findings complement the observed mitochondrial dysfunction and ATP5F1A depletion, which likely impair ATP-dependent transporters, such as Na^+^/K^+^-ATPase and Ca^2+^-ATPase, resulting in intracellular Na^+^ and Ca^2+^ overload, delayed repolarization, and increased arrhythmogenic potential [47,48]. Persistent NF-κB activation may further sustain mitochondrial and inflammatory dysfunction [49], forming a self-reinforcing cycle of energetic and electrical instability [50]. Overall, these data support that remdesivir-induced cardiotoxicity may arise from the interplay among inflammation activation, mitochondrial impairment, and ionic dysregulation. Careful cardiac monitoring is warranted during remdesivir therapy, particularly in male patients or patients with preexisting mitochondrial dysfunction.

The intraperitoneal administration of remdesivir induces whole-body drug exposure, and atrial pathological alterations, including inflammatory activation and mitochondrial dysfunction, are representative of the shared pathological state of the heart [51]. Systemic inflammation and oxidative stress can impair intercellular coupling via connexin 43 (Cx43) in both the atrial and ventricular myocardium. Evidence obtained from previous literature has indicated that TNF-α and IL-6 directly inhibit Cx43-mediated gap junction communication, and that mitochondrial ROS can induce Cx43 remodeling [52,53]. Cx43 dysfunction is a well-established substrate for slowed conduction and reentry, which underlies ventricular arrhythmias. Thus, remdesivir-induced systemic inflammation and oxidative stress may lead to Cx43 dysfunction in both the atrial and ventricular myocardium, resulting in local conduction slowing and increased heterogeneity, which in turn enhances the susceptibility to reentrant ventricular tachycardia.

Several limitations should be acknowledged in the present study. First, although guinea pigs share key similarities with human cardiac physiology, this model may not fully reflect the complexity of remdesivir-induced cardiotoxicity in patients. The molecular mechanisms underlying sex-specific differences, particularly those that involve mitochondrial fusion and fission, sex hormonal regulation, and genetic factors, remain to be explored. Future studies should explore these pathways and evaluate mitochondria-targeted interventions to mitigate remdesivir-associated cardiac injury. Second, although the observed reduction in ATP5F1A levels is consistent with mitochondrial impairment, ATP depletion is a relatively non-specific indicator, and its reduction may result from multiple mechanisms, including impaired mitochondrial oxidative phosphorylation, increased ATP consumption, or alterations in glycolytic pathways. In order to more directly confirm the mitochondrial dysfunction, future studies should include measurements of the NAD^+^/NADH ratio to evaluate the redox state and functional integrity of the electron transport chain, as well as assessments of mitochondrial respiratory chain complex activity, mitochondrial membrane potential, and ROS production. In addition, the analysis of mitochondrial dynamics-related proteins, such as fission and fusion regulators Drp1 and Mfn1/2, may help clarify the molecular mechanisms underlying mitochondrial structural and functional alterations. In addition, the QTc intervals in the present study were calculated using Bazett’s formula, which may overestimate QT prolongation at the relatively high HRs of guinea pigs. Future studies that employ species-specific correction formulas optimized for guinea pig HRs, such as the van de Water formula [54], may provide more accurate quantification. Lastly, the ventricular tissues were not directly analyzed. Thus, the link between atrial inflammation and ventricular arrhythmias remains to be confirmed.

## 4. Materials and Methods

### 4.1. Animal Model and Drug Administration

A total of 32 adult guinea pigs (16 male and 16 female guinea pigs, body weight: 250–350 g) were used for the present study. All animals underwent a 7-day acclimatization period under standardized housing conditions, including controlled temperature, humidity, and a fixed 12 h light/dark cycle. A stratified randomization design was employed to ensure sex balance. The animals were initially stratified by sex (16 male and 16 female animals), and subsequently randomly assigned to either the control group or model group using a random number table. Each group consisted of 16 animals (eight male and eight female animals). The control group received normal saline, while the model group was treated with remdesivir. All experimental procedures were approved by the Institutional Animal Care and Use Committee of Southwest Medical University, and conducted in accordance with the ARRIVE guidelines.

Due to the pronounced phenotypic changes during remdesivir treatment (i.e., reduced food intake, decreased activity, and diminished responsiveness), group allocation and drug administration were not performed in a blinded manner. However, echocardiographic image acquisition, parameter measurement, and data analysis were conducted by investigators blinded to the group assignment. The electrocardiography (ECG), optical mapping, enzyme-linked immunosorbent assay (ELISA), and Western blot measurements were recorded or analyzed automatically by the instrument or software, without additional blinding.

Remdesivir (Cat. no. R287694-1; Aladdin, Shanghai, China) was administered *via* intraperitoneal injection for five consecutive days. A loading dose of 23.6 mg/kg was given on day one, followed by a maintenance dose of 11.8 mg/kg from day two to five. The dosing regimen was derived from the clinically recommended human dosage, and converted to the equivalent animal dose based on body surface area to achieve stable systemic exposure. The drug was dissolved in a solvent mixture that consisted of 5% DMSO, 40% polyethylene glycol 300, and 55% sterile saline (0.9% NaCl), to a total injection volume of 1 mL. Then, the mixed solution was thoroughly vortexed, and ultrasonic dissolution was performed when necessary to ensure complete solubility. Afterwards, the solution was filtered through a 0.22 μm membrane for sterilization, and stored at 4 °C, protected from light until use.

Injections were performed at a 30° angle to avoid organ injury, and the abdominal area was gently massaged post-injection to promote drug dispersion. All injections were administered at a fixed time each day to minimize circadian variation. The animals were observed for an additional three days following the final administration to monitor recovery and delayed effects. After the experiments, the guinea pigs were anesthetized in a gas anesthesia machine with 3–4% isoflurane and subsequently euthanized by cervical dislocation.

### 4.2. Hematoxylin–Eosin (H&E) Staining

Atrial tissues were immediately excised after sacrifice, rinsed in sterile saline to remove residual blood, and fixed in 4% paraformaldehyde at room temperature for 24–48 h. Then, the fixed samples were dehydrated through a graded ethanol series (70%, 80%, 90%, 95% and 100%, each for 1–2 h), cleared twice in xylene (30–60 min each), and embedded in paraffin at 60 °C for 2–3 h. Afterwards, the paraffin-embedded blocks were sectioned at a thickness of 4–6 μm using a rotary microtome (model CM3050, Leica, Wetzlar, Germany). Subsequently, the sections were floated on a 40 °C water bath, mounted on glass slides, and baked at 60 °C for 1–2 h to enhance adhesion. Deparaffinization was performed sequentially in xylene I and II (15 min each), followed by rehydration through descending ethanol concentrations (100%, 95%, 85% and 75%, five minutes each). After rinsing in running water, the sections were stained with hematoxylin for 3–5 min, blued for 2–5 s, and counterstained with eosin for five minutes. The H&E staining reagents were purchased from ServiceBio (Cat. no. G1005, Wuhan, China). Next, the slides were dehydrated in graded ethanol, cleared twice in xylene for five minutes each, and mounted using neutral resin. The pathological evaluation was conducted under a light microscope (Model: MSCP1-40; Olympus, Tokyo, Japan), and digital images were captured for the analysis of myocardial architecture, inflammatory infiltration, and cellular necrosis.

### 4.3. Transmission Electron Microscopy (TEM)

For the ultrastructural analysis, the guinea pigs were anesthetized with 2–3% isoflurane delivered in oxygen until loss of reflexes. Then, the atrial tissue samples (~2 × 2 mm) were excised within 1–3 min (EM UC7 Ultramicrotome; Leica Microsystems, Wetzlar, Germany) after death. Afterwards, the tissues were immediately immersed in electron microscopy fixation buffer (Cat. no. G1102, ServiceBio) at room temperature for two hours, and stored at 4 °C. When rapid sampling was not feasible, preliminary fixation for 30 min was performed before trimming the tissue to the final dimensions. The samples were processed following standard TEM protocols, which included post-fixation, dehydration, embedding, and ultrathin sectioning. The mitochondrial morphology, cristae integrity, and presence of autophagic structures were evaluated by TEM (HT 7700 transmission electron microscope; Hitachi High-Tech, Tokyo, Japan).

### 4.4. Enzyme-Linked Immunosorbent Assay (ELISA)

The atrial tissue samples were rinsed with phosphate-buffered saline (PBS; 0.01 M, pH 7.4) to remove the surface blood and debris. After weighing, the tissues were minced and homogenized in ice-cold PBS that contained protease inhibitors at a 1:9 (*w*/*v*) ratio. Then, the homogenates were centrifuged at 5000× *g* for 10 min at 4 °C, and the supernatants were collected and stored at −80 °C until analysis. Commercial ELISA kits (Ruixin Biotechnology, Shanghai, China) were used to quantify the inflammatory cytokines, including interleukin-6 (IL-6; Cat. no. RX203049M), tumor necrosis factor-α (TNF-α, Cat. no. RX202412M), and nuclear factor kappa B (NF-κB; Cat. no. RX202896M), according to manufacturers’ protocols. Then, the adenosine triphosphate synthase F1 subunit alpha (ATP5F1A) content was measured (Cat. no. RXWB0028-96) as a marker of myocardial energy metabolism.

### 4.5. Western Blot

Atrial tissues were homogenized in lysis buffer under liquid nitrogen, and the total protein was extracted by centrifugation. Then, the protein concentrations were determined by bicinchoninic acid (BCA) assay (Beyotime, Shanghai, China). Equal amounts of protein (20–50 μg per lane) were mixed with sample loading buffer (4:1 ratio), and denatured at 100 °C for 5–10 min. Then, the proteins were separated by sodium dodecyl sulfate–polyacrylamide gel electrophoresis (SDS-PAGE), and transferred onto polyvinylidene fluoride (PVDF) membranes using a wet transfer system (200–300 mA, 60–90 min at 4 °C). Afterwards, the membranes were blocked with 5% non-fat milk in TBST for one hour (5% BSA for phospho-proteins) and incubated with primary antibodies (1:1000 dilution) against ATP (Cat. no. DF12111; Affinity Biologicals, Ancaster, ON, Canada), IL-6 (Cat. no. EM1701-45; HUABIO, Hangzhou, China), TNF-α (Cat. no. AF7014, HUABIO), and NF-κB (Cat. no. AF2006, Affinity Biologicals) overnight at 4 °C. After washing, horseradish peroxidase (HRP)-conjugated secondary antibodies (1:5000–1:10,000 dilution) were applied for one hour at room temperature. Then, the bands were visualized by enhanced chemiluminescence and quantified using ImageJ or Image Lab software (Version 1.54). The relative protein expression levels were normalized to internal controls.

### 4.6. In Vivo Electrocardiography (ECG) Recording

Continuous ECG recordings were obtained using a wireless telemetry system (Shanghai Kexin Technology Co., Ltd., Shanghai, China). The guinea pigs were acclimated for seven days prior to surgery. After 12 h fasting (with free access to water), anesthesia was induced and maintained with isoflurane (4% induction, 1.5–2.0% maintenance). Under aseptic conditions, telemetry electrodes were subcutaneously implanted on the dorsal surface and secured before closure. The animals were allowed to recover for three days before ECG monitoring. Recordings were obtained at three time points: baseline (24 h, pre-administration), day five (cumulative effect), and day eight (three days, post-treatment, recovery phase). The ECG parameters, which included RR interval, P-wave duration, QRS complex duration, QT interval, and corrected QT interval (QTc), were analyzed using the Kubios HRV software 4.1. The QT interval was corrected for heart rate (HR) using Bazett’s formula (QTc = QT/√RR). Then, the sex-dependent electrophysiological differences were evaluated.

### 4.7. Echocardiographic Assessment

Cardiac function was evaluated by transthoracic echocardiography using a high-frequency ultrasound imaging system (Vevo 3100; FUJIFILM VisualSonics Inc., Toronto, ON, Canada) equipped with a 15–30 MHz transducer. The guinea pigs were lightly anesthetized with 1.5–2.0% isoflurane to maintain stable respiration and HR during imaging. Then, standard parasternal long- and short-axis views were obtained, and the M-mode tracings were recorded at the papillary muscle level. Afterwards, parameters, which included ejection fraction (EF), fractional shortening (FS), and interventricular septal thickness during diastole and systole (IVS;d and IVS;s), were measured over three consecutive cardiac cycles, and averaged. All measurements were analyzed using the manufacturer’s software by an investigator blinded to the group allocation.

### 4.8. Ex Vivo ECG Recording

The ex vivo cardiac electrophysiology was assessed using the Langendorff perfusion system (Model: LGF-2C; Asinstrument, Dallas, TX, USA). The animals were pretreated with heparin (0.0065 mL per g body weight, 500 U/mL) via intraperitoneal injection at 20 min prior to euthanasia, in order to ensure effective anticoagulation. Following euthanasia, the hearts were rapidly excised and perfused at 37 °C with the oxygenated Krebs–Henseleit solution (118 mM of NaCl, 4.7 mM of KCl, 1.8 mM of CaCl_2_, 1.2 mM of MgSO_4_, 25 mM of NaHCO_3_, and 11 mM of glucose). After stabilization, the hearts were exposed to remdesivir (Cat. no. HY-104077; MedChemExpress, Monmouth Junction, NJ, USA) at concentrations of 3 μM and 10 μM, and the ECG changes were recorded. Then, the RR, PR, QT, and QTc intervals, as well as the ventricular tachycardia (VT) episodes, were analyzed to evaluate the electrophysiological impact of remdesivir. The QT interval was corrected for HR using Bazett’s formula (QTc = QT/√RR).

### 4.9. Optical Mapping

The optical mapping of isolated guinea pig hearts was conducted using RH237 voltage-sensitive dye (Cat. no. sc-499456; Santa Cruz Biotechnology, Dallas, TX, USA). Following Langendorff perfusion and stabilization, cardiac motion was suppressed using a cardioplegic agent. Then, the hearts were perfused with RH237-containing KH solution for 15 min, ensuring complete dye incorporation. Optical recordings were obtained under controlled illumination conditions at baseline, at 3 μM, and 10 μM of remdesivir (Cat. no. HY-104077, MedChemExpress) exposure, and at the drug washout phases. Parameters, which included activation time, action potential duration at 50% (APD50) and 80% repolarization (APD80), and conduction velocity (CV), were analyzed. All experiments were performed under constant temperature and perfusion conditions to minimize variability.

During the optical mapping recordings, programmed cardiac electrical stimulation was applied synchronously to assess the arrhythmias. Arrhythmic events were induced using a high-frequency S1-S1 pacing protocol at 20 Hz to detect and induce reentrant activity. The stimulation cycle length was set at 200 ms, and the stimulation intensity was adjusted to1.5–2.0 times the pacing threshold.

The analysis was performed using ElectroMap 1.0, which is an open-source electrophysiological data analysis platform developed in MATLAB (Matlab2019, Mathworks, Natick, MA, USA).

### 4.10. Statistical Analysis

The sample size was determined based on preliminary experiments, previously published studies on drug-induced cardiac toxicity in guinea pigs, and the anticipated mortality rate [55,56,57]. Accordingly, eight animals per group (*n* = 8) were initially included to ensure a final effective sample size of at least six animals per group (*n* ≥ 6) for the statistical analysis. Six animals were randomly selected from each group. Thus, During the experimental period, one male guinea pig in the model group died on day five due to drug intolerance, exhibiting reduced food intake, decreased activity, and diminished responsiveness, and was thereby excluded from all analyses. The remaining 31 animals completed the study. For the final statistical analysis, six animals were randomly selected from each group. Thus, the final effective sample size in each group met the predefined statistical requirement.

GraphPad Prism 10 and Fiji (ImageJ 2.16.0) 1.54p were used for the data analysis and visualization. All data were presented in mean ± standard deviation. The normality of data distribution was assessed using the Shapiro–Wilk test, and the homogeneity of variances was evaluated using Levene’s test. Comparison between groups was performed using two-way analysis of variance (ANOVA) with Šídák’s post hoc analysis. For the in vivo ECG comparison, repeated-measures ANOVA were performed. When the sphericity assumption was violated, Greenhouse–Geisser correction was applied. Šídák’s multiple-comparison test was used for post hoc comparisons. A *p*-value of <0.05 was considered statistically significant.

## 5. Conclusions

The present study revealed that remdesivir-associated cardiac effects are greater in males, with more pronounced myocardial inflammation, mitochondrial dysfunction, and electrophysiological disturbances, including prolonged action potentials and arrhythmias. These results provide mechanistic insights into the remdesivir-related cardiotoxicity and highlight the importance of considering sex as a critical factor in clinical risk assessment. Clinically, the present findings support the close monitoring of cardiac function, particularly in male patients receiving remdesivir, and the integration of sex-specific strategies in managing drug-induced cardiac adverse effects during COVID-19 treatment.

## Data Availability

The data presented in this study are available on request from the corresponding author.
